# Pulmonary alveolar proteinosis with upper‐lobe predominance in a non‐smoking female

**DOI:** 10.1002/rcr2.445

**Published:** 2019-05-29

**Authors:** Hong‐Ping Er, Chung‐Ta Lee, Tang‐Hsiu Huang

**Affiliations:** ^1^ Division of Chest Medicine, Department of Internal Medicine, National Cheng Kung University Hospital, College of Medicine National Cheng Kung University Tainan Taiwan; ^2^ Department of Pathology, National Cheng Kung University Hospital, College of Medicine National Cheng Kung University Tainan Taiwan

**Keywords:** Crazy‐paving pattern, ground‐glass opacity, pulmonary alveolar proteinosis, upper‐lobe predominance

## Abstract

In this report, we describe an unusual manifestation of pulmonary alveolar proteinosis (PAP). The patient is a 43‐year‐old non‐smoking female without underlying hematologic or auto‐immune disorder. Her initial presentation included non‐specific respiratory symptoms (exertional dyspnoea and cough), an unremarkable physical examination, a mild elevation in her serum level of lactate dehydrogenase, a mild impairment in the diffusion capacity for carbon monoxide but a normal spirometry, and multiple ground‐glass opacities with a “crazy‐paving” pattern predominantly in upper lung zones on her chest radiographic images. PAP was diagnosed histologically. PAP commonly occurs in males with smoking history, and tends to affect the lung parenchyma diffusely or, as in auto‐immune PAP, lower lobes predominantly. Upper‐lobe predominant PAP, particularly in a non‐smoking female, is rare. This report would add PAP to the list of differential diagnosis for upper‐lung ground‐glass opacities. A review on the relevant literature is also included in the discussion.

## Introduction

Pulmonary alveolar proteinosis (PAP) is a diffuse parenchymal lung disease characterized by the accumulation of amorphous, periodic acid–Schiff (PAS)‐positive lipoproteinaceous material in distal air spaces. It is more common in male than in female, particularly those with a positive smoking history. Typical chest X‐ray (CXR) shows symmetric and centrally located alveolar opacities in bilateral mid‐ and lower‐lung zones. In this report we describe a non‐smoking young female who developed histologically proven PAP with an unusual upper‐lobe predominance. The relevant literature is also reviewed.

## Case Report

**Figure 1:**
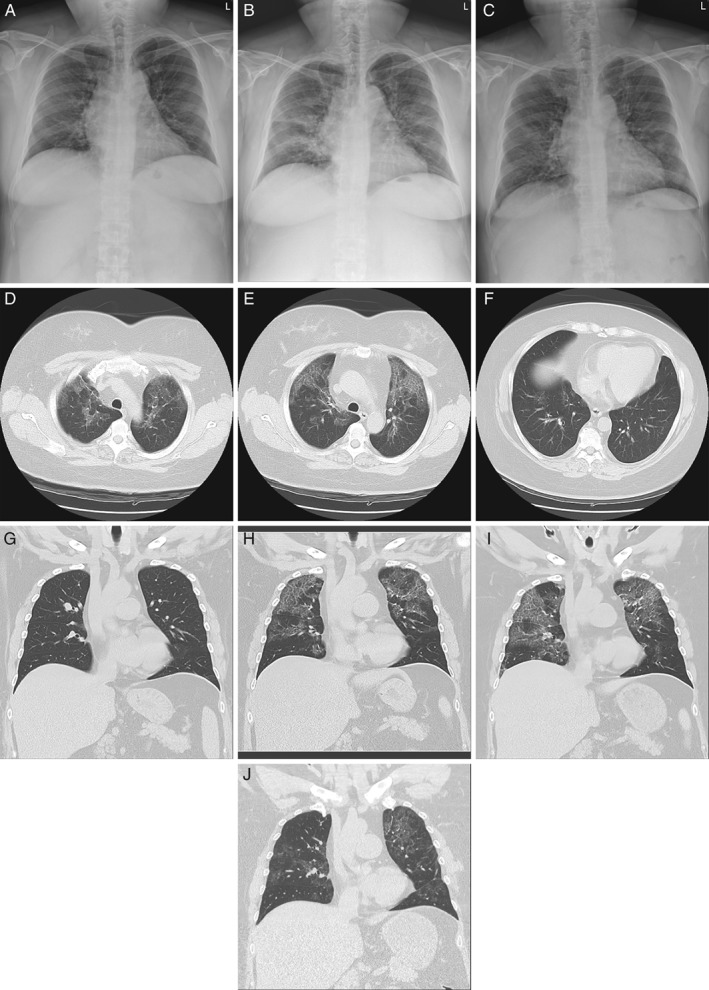
Serial radiographic images of the patient. Postero‐anterior chest roentgenograms taken one year earlier (A), at initial presentation (B), and 17 months later (C); computed tomography scan images: transverse sections at different levels (D–F) at initial presentation, and coronal views one year earlier (G), at initial presentation (H), six months later (I), and 17 months later (J).

**Figure 2:**
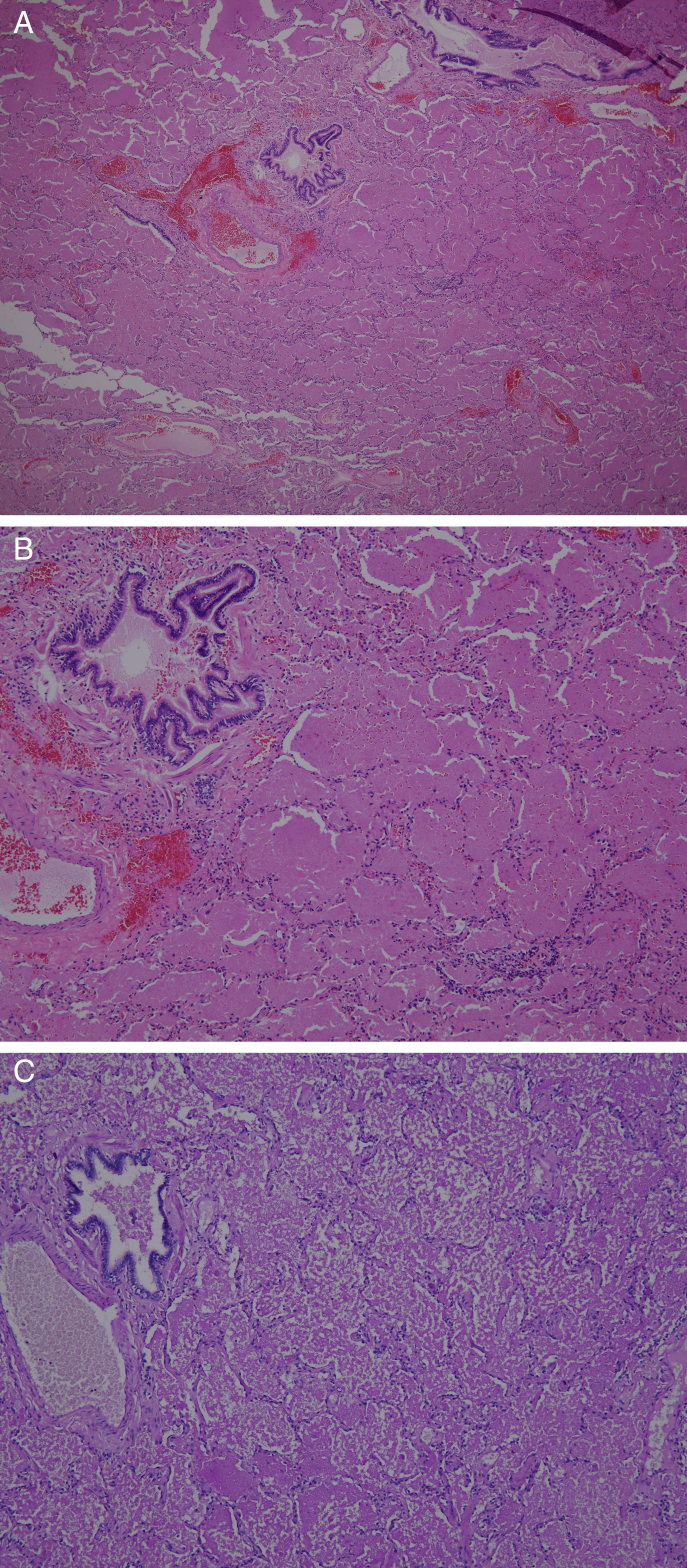
Under microscopy, the alveolar architectures appeared well preserved, but the alveolar space was extensively filled with acellular pinkish exudate that stained positive for periodic acid–Schiff (PAS) stain. (A, Hematoxylin and eosin (H&E) stain at 40× original magnification; B, H&E stain at 100× original magnification; C, PAS stain at 100× original magnification).

A 43‐year‐old non‐smoking female presented to our chest medicine clinic with mild exertional dyspnoea and dry cough for 1 month. There was no associated fever, chest pain, orthopnoea, paroxysmal nocturnal dyspnoea, dysphagia, abdominal pain, arthralgia, weakness of limbs, or skin eruption. Her past medical history was positive for obesity and type 2 diabetes mellitus, while her health status during childhood and adolescence, and her occupational and exposure histories, were unremarkable. Upon physical examination, auscultation of her chest detected enhanced bronchial sound diffusely in her lung fields. Her body temperature was normal. The rest of the examination was unremarkable. However, her CXR showed new patchy and linear infiltrates mainly in the peri‐hilar and upper fields as compared with the film taken about one year ago (Fig. 1A, B). The subsequent computed tomography (CT) of the chest revealed multiple well‐delineated opacities with a “crazy‐paving” pattern predominantly involving her apical and upper lungs (Fig. 1D–F, H). Notably, these opacities were absent from her past radiographic images (Fig. 1A, G). The pulmonary function test reported normal lung volume and spirometry, but a mild impairment in the diffusion capacity for carbon monoxide (DLco; 64%). No significant structural anomaly or ventricular dysfunction was detected by the echocardiography. At first the patient declined any further invasive investigation and thus received a regular follow‐up. Over the next 3 months, her symptoms and radiographic findings had remained stable. After thorough discussion, the patient finally agreed with invasive diagnostic studies for her lung lesions. Analysis of the bronchoalveolar lavage (BAL) fluid reported a turbid appearance but the presence of only few white blood cells (30/mm^3^, predominantly macrophages). Cultures of the lavage fluid yielded negative growth of any microbe, while the cytology reported no evidence of malignant cells. Due to the undiagnostic BAL, video‐assisted thoracoscopic wedge‐biopsy of her left‐upper‐lobe lung was subsequently performed. Microscopic examination of the biopsied tissue observed a well‐preserved alveolar architecture with extensive acellular pinkish exudate in the alveolar space that stained positive for PAS (Fig. 2A–C). There was no evidence of microbes, including *Pneumocystis carinii*. The diagnosis of PAP was established histologically. Granulocyte‐macrophage‐colony stimulating factor (GM‐CSF) auto‐antibody could not be checked due to the unavailability at our institute, whereas serological tests for other auto‐antibodies were all negative (Table 1). Complete peripheral blood cell count showed no evidence of anaemia, hematologic malignancy, or myelodysplastic syndrome. Nevertheless, her serum level of lactate dehydrogenase (LDH) was mildly elevated (276 U/L; normal range, 135–225 U/L). The patient since then has been regularly followed at our hospital. The most recent CXR showed significant resolution of the infiltrates (Fig. 1C, taken about 17 months after that in Fig. 1A). Serial follow‐up CT scans of the chest revealed migratory changes in those lung opacities, with dynamic antero‐posterior shifts in her upper‐lobe lesions, and fluctuating patches in her lower‐lung zones (Fig. 1H–J). Serial pulmonary function tests revealed a declining trend in her forced vital capacity, and a mild but persistent impairment in her DLco (Table 2). Meanwhile, her symptoms have remained relatively mild and stable. Her serum level of LDH checked one year later has returned to the normal range (Table 1).

**Table 1:** Relevant blood biochemical and serological tests of the patient.

Auto‐antibody	Serum level or titre	Normal range (unit)
Antinuclear antibody	1:40	<1:160
Anti‐dsDNA Ab	1:10	≤1:10
Anti‐U1‐RNP Ab	0.4	<5 (U/mL)
Anti‐SS‐A/Anti‐Ro Ab	1.7	<7 (U/mL)
Anti‐SS‐B/Anti‐La Ab	0.1	<7 (U/mL)
Anti‐Sm Ab	9.8	<10 (U/mL)
Anti‐topoisomerase/Anti‐Scl‐70 Ab	2.2	<7 (U/mL)
Anti‐Jo 1 Ab	0.1	<7 (U/mL)
C3	156.0	58.0–147.0 (mg/dL)
C4	39.7	11.0–35.0 (mg/dL)
HIV screening test	Negative	Negative
LDH (3 January 2018)	276	135–225 (U/L)
LDH (13 January 2019)	213	135–225 (U/L)

Ab, antibody; HIV, human immunodeficiency virus; LDH, lactate dehydrogenase.

**Table 2:** Measurements of serial pulmonary function tests of the patient.

Date of tests	26 June 2016	29 December 2017	21 June 2018	26 November 2018
FVC, L (%pred)	3.00 (106)	2.68 (96)	2.55 (93)	2.62 (95)
FEV_1_, L (%pred)	2.51 (95)	2.31 (90)	2.21 (88)	2.32 (92)
FEV_1_/FVC, %	84	86	87	89
TLC, L (%pred)	NA	3.84 (92)	NA	NA
DLco, %pred	NA	64	61	65

%pred, percentage of the predicted value; DLco, diffusion capacity for carbon monoxide; FEV_1_, forced expiratory volume in 1 s; FVC, forced vital capacity; NA, not available; TLC, total lung capacity.

## Discussion

PAP (also known as pulmonary alveolar phospholipoproteinosis) is a rare lung disease characterized by the accumulation of amorphous, PAS‐positive lipoproteinaceous material in the alveola with little or no inflammation and well‐preserved lung architectures 1, 2, 3, 4, 5. Unlike disorders of surfactant production, in PAP the synthesis of surfactants is normal, while the main disorder lies in the clearance of surfactants, for which alveolar macrophages and the cytokine GM‐CSF play critical roles 3. PAP can be classified into hereditary and non‐hereditary. Hereditary PAP is caused by mutations in genes encoding subunits of GM‐CSF receptors 6, 7, 8. For non‐hereditary PAP, most cases are due to the presence of anti‐GM‐CSF auto‐antibodies that disrupt the signalling of the cytokine (“auto‐immune PAP”) 9, 10, while the rest have “secondary PAP” that is either triggered by extrinsic stimuli (such as particles and fumes) or caused by an underlying condition (such as infection or hematologic disorders) that impairs the number or function of alveolar macrophages 2, 3, 4, 5, 6, 11, 12.

Non‐hereditary PAP typically occurs between the fifth and sixth decade of age. It is about two‐fold more common in male than in female, and is more common in smokers or those with a positive history of causative exposure. The onset is usually insidious, with such non‐specific symptoms as dyspnoea, cough, fatigue, and occasionally fever 10, 12, 13, 14, 15, 16, 17, 18, 19, 20. Pulmonary function tests almost consistently reveal a reduction in the DLco indicating the presence of ventilation‐perfusion mismatch, with or without a restrictive‐type ventilatory deficit 2, 4, 5. Typical radiographic findings of PAP include bilateral symmetric alveolar opacities or even consolidation that are located centrally, often in a “bat wing” distribution, on the postero‐anterior chest roentgenogram, although a predominantly reticular pattern has also been reported 21, 22. Pleural effusion is typically absent. CT scan (particularly high‐resolution CT) generally reveals bilateral ground‐glass opacities with a geographical distribution that are superimposed by thickened intra‐ and interlobular septa, which altogether constitute the typical “crazy‐paving pattern” 23, 24, 25. Overall these opacities most frequently involve either the entire lung diffusely or predominantly the mid‐to‐lower‐lung zones 4, 5, 13, 21, 23, 24. Holbert et al. reviewed 139 CT scans from 27 patients with PAP and found that, depending on whether a thick‐ or thin‐section scanning mode was applied, opacities on 63.3% to 65.2% of scans exhibited a diffuse distribution, 21.7 to 23.4%, a lower‐lobe predominance, and 8.2% to 13.3%, an upper‐lobe predominance 24. Frazier et al. reviewed the radiographic images of 98 patients with PAP (including 89 CXRs and 28 CT scans) from a well‐established archive. They found that 44%, 27%, 25%, and 5% of patients had mid‐lung‐predominant, diffuse, lower‐predominant, and upper‐predominant opacities on the CXRs, respectively; 71%, 14%, and 14% of patients had diffuse, lower‐predominant, and upper‐predominant opacities on the CT scans, respectively 21. It has also been reported that compared to secondary PAP in which radiographic opacities tend to exhibit a diffuse distribution, opacities in auto‐immune PAP more frequently show a lower‐lobe predominance 26. The predilection of PAP for a diffuse or lower‐predominant involvement could be demonstrated even using ultra‐low‐dose CT scans 27. PAP with an isolated upper‐lobe predominance is relatively rare. BAL fluid recovered from affected lung segments or lobes may show an opaque or even milky appearance due to the presence of abundant lipoproteinaceous material that precipitates upon standing 28. Papanicolaou‐stain of the lavage fluid may aid in the diagnosis of PAP 29. A definitive diagnosis of PAP, however, requires lung tissue sampling and the histological evidence of intra‐alveolar accumulation of PAS‐positive lipoproteinaceous material with grossly normal alveolar architecture and minimal or no associated inflammation 2, 4, 5, 21. Serum biomarkers other than anti‐GM‐CSF auto‐antibody (such as LDH, surfactant protein A and D, Krebs von den Lungen‐6), though neither specific nor diagnostic, may be used to follow the disease activity of PAP 5, 10.

Treatment for PAP depends on the severity of disease. Removal and avoidance of known causative exposure is particularly critical for managing secondary PAP. Patients who have asymptomatic and mild disease can receive symptomatic treatment and close observation. Spontaneous improvement has been reported. For those with more advanced disease or even respiratory distress, whole‐lung lavage still remains the standard therapy 5, 30, 31, 32, 33. Novel therapies have been proposed and tested specifically for auto‐immune PAP, including GM‐CSF supplement (particularly delivered via inhalation), rituximab, and plasmapheresis 30, 31, 32, 34. Efficacy of these experimental therapies for secondary PAP is still not established. Lung transplantation may be considered in severe and refractory cases, though recurrence has been reported 35.

The diagnosis of PAP in our patient is based on histology and is therefore reliable. Considering that no underlying predisposing condition was identified, the patient probably had auto‐immune PAP. However, we could not check her serum level of anti‐GM‐CSF auto‐antibody. Moreover, although the patient had an uneventful growth and a near‐normal chest CT scan one year ago (Fig. 1A, G), without checking the genetic profiles of the patient and her parents, the possibility of hereditary PAP cannot be completely excluded. The unusual features of her disease include the female sex, the negative smoking history, and in particular the rare presentation of isolated upper‐lobe predominance. Another noteworthy manifestation of her PAP is the dynamic change in the zonal distribution of radiographic opacities. Spontaneous partial improvement or even resolution of PAP has been reported 33. Moreover, the dorso‐ventral migration of opacities on the CT scan, probably induced by the gravitational effect following an extended session of prone position, has also been well described 36. These peculiar features of PAP would need to be considered when planning invasive diagnostic procedure for tissue or lavage‐fluid sampling.

In conclusion, we report an unusual presentation of PAP that involved predominantly the upper lobes. This report would add PAP to the list of differential diagnosis when approaching patients with lung conditions manifesting as ground‐glass opacities in the upper‐lung zones.

### Disclosure Statements

Appropriate written informed consent was obtained for publication of this case report and accompanying images. Parts of the information described in this case report were presented in a thematic poster in the 23rd Congress of the Asian Pacific Society of Respirology, 2018.
